# PGC-1α Suppresses the Activation of TGF-β/Smad Signaling via Targeting TGFβRI Downregulation by *let-7b/c* Upregulation

**DOI:** 10.3390/ijms20205084

**Published:** 2019-10-14

**Authors:** Hoon-In Choi, Jung Sun Park, Dong-Hyun Kim, Chang Seong Kim, Eun Hui Bae, Seong Kwon Ma, Soo Wan Kim

**Affiliations:** Department of Internal Medicine, Chonnam National University Medical School, 42 Jebongro, Gwangju 61469, Korea; hoonin_c@hanmail.net (H.-I.C.); gene-pjs@hanmail.net (J.S.P.); dhkim450@gmail.com (D.-H.K.); laminion@hanmail.net (C.S.K.); baedak76@gmail.com (E.H.B.); drmsk@hanmail.net (S.K.M.)

**Keywords:** PGC-1α, *let-7b/c* miRNA, TGFβRI, TGF-β/Smad signaling, epithelial-mesenchymal transition (EMT)

## Abstract

TGF-β/Smad signaling is a major pathway in progressive fibrotic processes, and further studies on the molecular mechanisms of TGF-β/Smad signaling are still needed for their therapeutic targeting. Recently, peroxisome proliferator-activated receptor γ coactivator-1α (PGC-1α) was shown to improve renal fibrosis, making it an attractive target for chronic kidney diseases (CKDs). Here, we show the mechanism by which PGC-1α regulates the TGF-β/Smad signaling pathway using HK-2 cell lines stably overexpressing empty vector (mock cells) or *human PGC1α* (PGC1α cells). Stable PGC-1α overexpression negatively regulated the expression of TGF-β-induced epithelial-mesenchymal transition (EMT) markers (fibronectin, E-cadherin, vimentin, and α-SMA) and EMT-related transcription factors (Snail and Slug) compared to mock cells, inhibiting fibrotic progression. Interestingly, among molecules upstream of Smad2/3 activation, the gene expression of only TGFβRI, but not TGFβRII, was downregulated in PGC-1α cells. In addition, the downregulation of TGFβRI by PGC-1α was associated with the upregulation of *let-7b/c*, miRNA for which the 3′ untranslated region (UTR) of TGFβRI contains a binding site. In conclusion, PGC-1α suppresses TGF-β/Smad signaling activation via targeting TGFβRI downregulation by *let-7b/c* upregulation.

## 1. Introduction

Renal fibrosis is a central event in the progression of chronic kidney diseases (CKDs) that leads to end-stage kidney diseases [1]. Among multiple mediators, transforming growth factor-β (TGF-β) is a key mediator that triggers activation of progressive renal fibrosis signaling pathways [1,2]. These pleiotropic effects of TGF-β are mediated via three types of TGF-β receptors (TGFβRI, TGFβRII and TGFβRIII) that function as serine-threonine kinases. In a Smad-dependent pathway, TGF-β binds to TGFβRII, which binds and phosphorylates TGFβRI. These events trigger the recruitment of the receptor-regulated Smad proteins (R-Smads) Smad2 and Smad3 to the cytoplasmic domain of activated TGFβRI, which then phosphorylates Smad2/3. Once phosphorylated, Smad2/3 form a trimer with Smad4, which is then translocated to the nucleus where it binds to Smad-binding elements to modulate target genes [3]. Targeting TGF-β/Smad signaling remains an attractive target for the development of therapeutics for fibrotic progression [4].

PGC-1α is a master regulator of mitochondrial biogenesis [5]. Mitochondria are a major energy source for cells, and the kidney requires a constant level of ATP to transport solutes along nephrons. Indeed, the expression of PGC-1α in the kidney overlaps in the proximal tubule and medullary thick ascending limb of Henle, where mitochondrial activity is high [6]. Many studies have suggested that PGC-1α is an attractive candidate that plays a role in kidney chemoprevention by improving CKD [7]. The expression of PGC-1α was significantly reduced in not only kidney biopsy specimens derived from CKD patients but also unilateral ureteral obstruction-, folic acid- and APOL1-induced fibrosis models [8,9,10]. Proximal tubule-specific deletion of liver kinase B1 (*LKB1*), an upstream regulator of PGC-1α, resulted in augmented renal fibrosis [11]. In addition, Notch1/HES1-mediated PGC-1α downregulation was reported, and tubule-specific PGC-1α overexpression in a Notch-induced fibrosis mouse model alleviated profibrotic gene expression and renal fibrosis [8]. Taurine upregulated gene 1 (*Tug1*), a conserved long noncoding RNA, positively regulates PGC-1α, which has a *Tug1*-binding element in its upstream promoter, leading to increased PGC-1α transcriptional activity. In diabetic nephropathy (DN), podocyte-specific overexpression of *Tug1* improved low PGC-1α expression, and the direct interaction between *Tug1* and PGC-1α improved DN-related biochemical and histological features [12]. However, the molecular basis by which PGC-1α regulates profibrotic gene expression and fibrotic signal pathway remains to be further elucidated.

In this study, we aimed to determine whether PGC-1α can regulate TGF-β/Smad signaling, a core pathway in fibrotic progression and, if so, what the regulatory mechanism of this effect is. We found that the overexpression of PGC-1α negatively regulated the expression of TGFβRI through the downregulation of TGFβRI due to PGC-1α-mediated *let-7b/c* upregulation.

## 2. Results

### 2.1. Downregulation of PGC-1α in UUO-Induced Kidney Injury and TGF-β-treated HK-2 Cells

**Figure 1:**
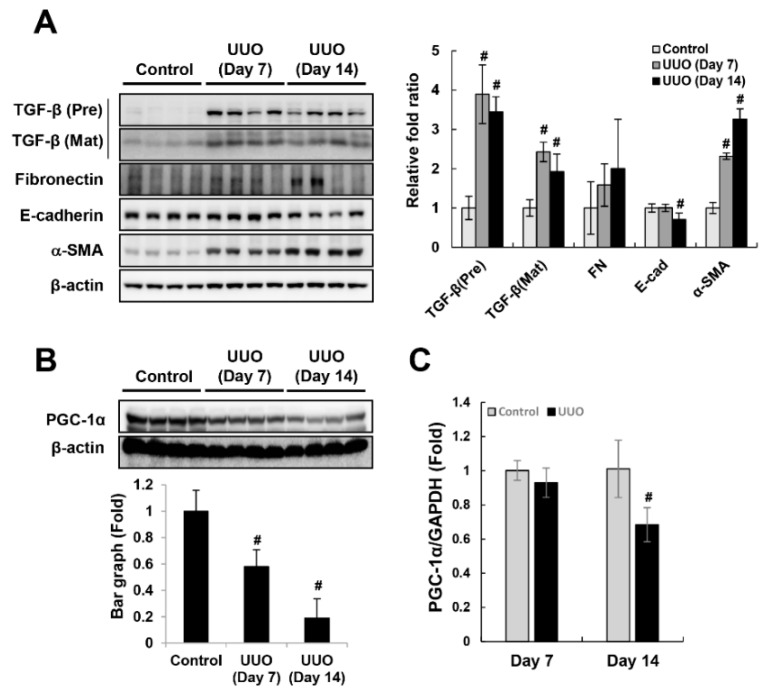
Downregulation of PGC-1α in the unilateral ureteral obstruction (UUO)-induced fibrotic kidney. To assess the fibrotic progression in the left kidney during ureter obstruction, we analyzed the expression of fibrotic marker proteins (premature and mature forms of TGF-β, fibronectin, and α-SMA) and an epithelial marker protein (E-cadherin) (**A**). To assess the involvement of PGC-1α in fibrotic progression, we analyzed the protein (**B**) and mRNA (**C**) levels of PGC-1α in whole-kidney homogenates. Bar graphs of protein levels show mean PGC-1α/β-actin expression, as measured by densitometry, and bar graphs of mRNA levels show mean PGC-1α/GAPDH expression. **^#^** *p* < 0.05, day 7 and day 14 UUO kidney vs. control kidney.

**Figure 2:**
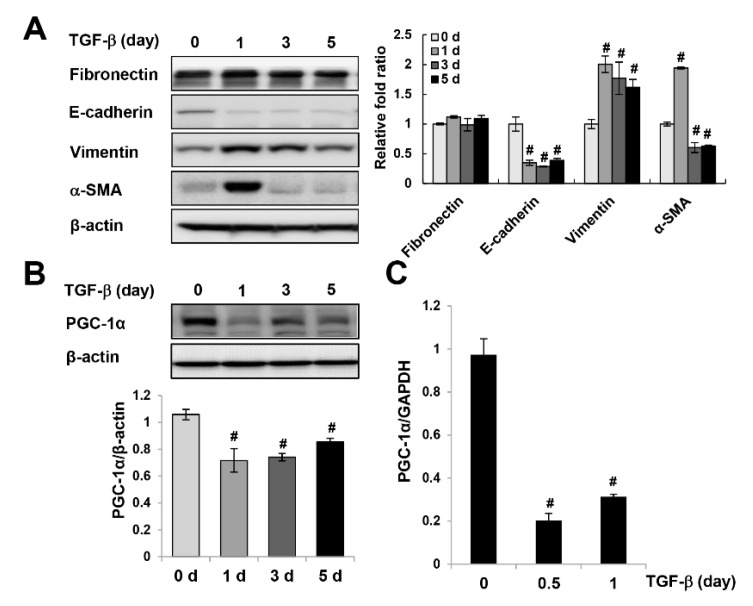
Downregulation of PGC-1α in TGF-β-treated HK-2 cells. HK-2 cells were exposed to TGF-β. The protein expression of epithelial-mesenchymal transition (EMT) markers (fibronectin, E-cadherin, vimentin, and α-SMA) (**A**) and PGC-1α (**B**) was analyzed by western blotting. Bar graphs show mean PGC-1α, fibronectin, E-cadherin, vimentin, and α-SMA/β-actin expression levels, as measured by densitometry. (**C**) The mRNA expression levels of PGC-1α in TGF-β-treated HK-2 cells were analyzed by real-time polymerase chain reaction (PCR). **^#^** *p* < 0.05, TGF-β-treated vs. untreated.

To clarify the physiological involvement of PGC-1α in fibrotic progression, we analyzed the expression pattern of PGC-1α in unilateral ureteral obstruction (UUO)-induced kidney injury and TGF-β-treated HK-2 cells. As the left ureteral ligation was performed for 7 days (*n* = 8) and 14 days (*n* = 8), fibrotic progression was intensified. The expression of the fibrotic markers TGF-β (precursor form and mature form), fibronectin, and α-SMA increased gradually with fibrotic progression. The expression of E-cadherin, which is characteristic of epithelial cells, was reduced in the UUO kidney and accumulation of extracellular matrix protein (Col1a1 and Col3a1) was increased in the UUO kidney (Figure 1A and Figure S1). Furthermore, the protein and mRNA levels of PGC-1α declined inversely with fibrotic progression (Figure 1B,C). Consistent with the reduction in PGC-1α in UUO kidneys, the protein expression of PGC-1α in HK-2 cells was lowest at the same time when the TGF-β-induced fibrotic progression peaked after one day (Figure 2A,B). The mRNA expression of PGC-1α was also decreased by TGF-β treatment (Figure 2C). These results suggest that PGC-1α is a target protein that responds to fibrotic stress.

### 2.2. Protective Effects of PGC-1α on TGF-β-Induced Epithelial-Mesenchymal Transition (EMT)

**Figure 3:**
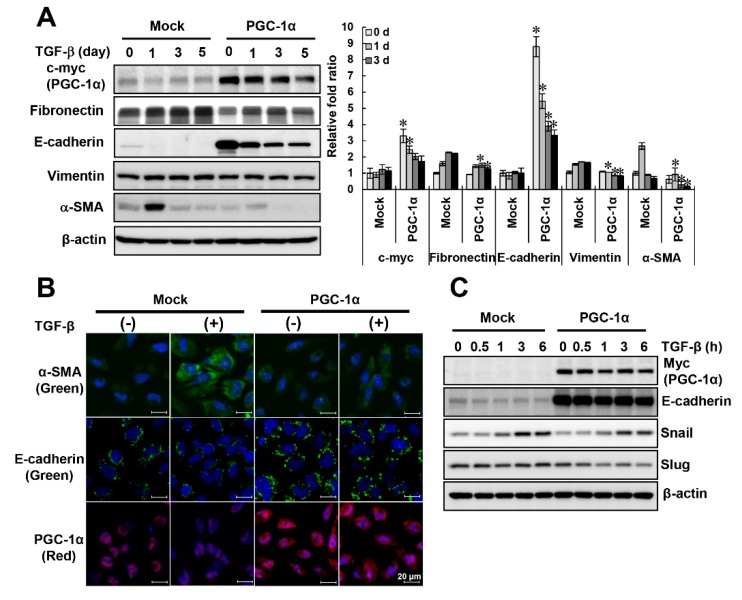
Comparison of EMT phenotype in TGF-β-treated mock or PGC-1α HK-2 cells. To confirm the protective effect of PGC-1α on EMT, HK-2 cells stably overexpressing PGC-1α (PGC-1α cells) and mock cells were treated with TGF-β and compared. (**A**) The protein expression levels of PGC-1α (c-myc tagged) and EMT markers (fibronectin, E-cadherin, vimentin, and α-SMA). (**B**) Morphological changes in PGC-1α (red) and EMT markers (green, labeled E-cadherin and α-SMA) by immunofluorescence staining (original magnification, 800×; bar = 20 μm). (**C**) Effects of changes in transcription factors (Snail and Slug) on E-cadherin expression in both cell lines were compared. Bar graphs show mean PGC-1α (c-myc-tagged), fibronectin, E-cadherin, vimentin, and α-SMA/β-actin expression levels, as measured by densitometry. * *p* < 0.05, mock cells vs. PGC-1α cells at the indicated time (0, 1, 3, and 5 days).

To examine the role and molecular mechanism of PGC-1α in fibrotic progression, we used an HK-2 cell line stably overexpressing *human PGC-1α* (PGC-1α cells) [13] and compared the expression patterns of fibrotic markers with those of mock cells following TGF-β treatment. Consistent with the data shown in Figure 2A, the protein expression of fibronectin, vimentin, and α-SMA in mock cells was increased by TGF-β treatment, while their expression in PGC-1α cells was significantly reduced. In particular, PGC-1α cells showed a marked increase in the expression of E-cadherin, which is characteristic of epithelial cells. In addition, the amount of E-cadherin in PGC-1α cells was much higher than that in mock cells, although E-cadherin levels were decreased by TGF-β treatment (Figure 3A). Alternatively, to confirm changes in EMT markers induced by PGC-1α, we performed immunofluorescence staining, with red fluorescence indicating PGC-1α and green fluorescence indicating α-SMA or E-cadherin. Green fluorescence indicating α-SMA was increased by TGF-β treatment in mock cells, but not PGC-1α cells. Green fluorescence indicating E-cadherin was also decreased in TGF-β-treated mock cells, but its high level in PGC-1α cells did not change much following TGF-β treatment (Figure 3B). The expression of Snail and Slug, a transcription factor that affects E-cadherin expression, in TGF-β-treated PGC-1α cells, was lower than that in TGF-β-treated mock cells (Figure 3C). These results confirmed that PGC-1α has an inhibitory effect on TGF-β-induced EMT.

### 2.3. Negative Regulation of TGF-β/Smad Signaling by PGC-1α

**Figure 4:**
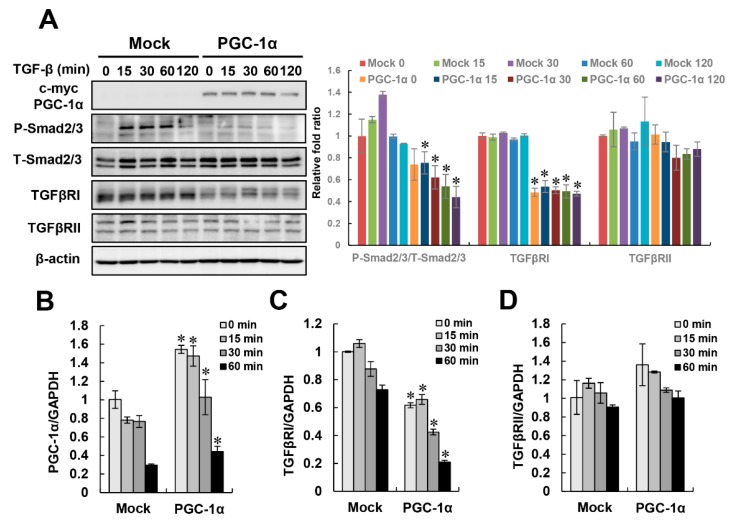
Inactivation of TGFβ/Smad signaling by PGC-1α. To identify signaling pathways related to the protective effect of PGC-1α on EMT, the regulation of canonical TGF-β/Smad signaling at the protein (**A**) or mRNA (**B**–**D**) levels was assessed in mock and PGC-1α cells at the indicated times (0, 15, 30, 60, and 120 min for protein levels or 0, 15, 30, and 60 min for mRNA levels) after TGF-β treatment. Protein expression and the activation of canonical TGF-β/Smad signaling components were analyzed by measuring Smad2 phosphorylation at Ser465/467 and Smad3 phosphorylation at Ser423/425. Total expression levels of Smad2/3, TGFβRI, and TGFβRII were evaluated with anti-Smad2/3, anti-TGFβRI, and TGFβRII antibodies, respectively. Bar graphs show mean ratios of the phosphorylated to total forms of Smad2/3, TGFβRI, and TGFβRII/β-actin, as measured by densitometry. The mRNA levels of PGC-1α, TGFβRI, and TGFβRII were analyzed by real-time PCR. Bar graphs show mean ratios of the indicated targets (PGC-1α, TGFβRI, and TGFβRII) to GAPDH, which was used as an internal reference. * *p* < 0.05, mock cells vs. PGC-1α cells at the indicated time.

We wondered what signal pathway is involved in the protective effect of PGC-1α on TGF-β-induced EMT. To address this question, we observed the activation of the canonical TGF-β/Smad signal pathway, which is a major signaling pathway in fibrotic progression. In mock cells, the phosphorylation of Smad2/3 rapidly increased after 15 min of treatment with TGF-β, while Smad2/3 phosphorylation was effectively inhibited in PGC-1α cells. Interestingly, the protein levels of TGFβRI, a molecule upstream of the activation of Smad2/3, were downregulated in PGC-1α cells. The protein expression of TGFβRII was not significantly changed in either mock or PGC-1α cells. To examine whether this decrease in the TGFβRI protein level by PGC-1α is regulated at the transcriptional or translational level, we examined time-dependent mRNA changes in both TGF-β-treated mock and PGC-1α cells. The increase of PGC-1α mRNA in PGC-1α cells caused a decrease in TGFβRI mRNA, and there was no significant effect on TGFβRII in in both TGF-β-treated mock and PGC-1α cells (Figure 4A–D). These results suggest that the downregulation of TGFβRI by PGC-1α is regulated at the transcriptional level.

### 2.4. PGC-1α-Specific TGFβRI Downregulation

**Figure 5:**
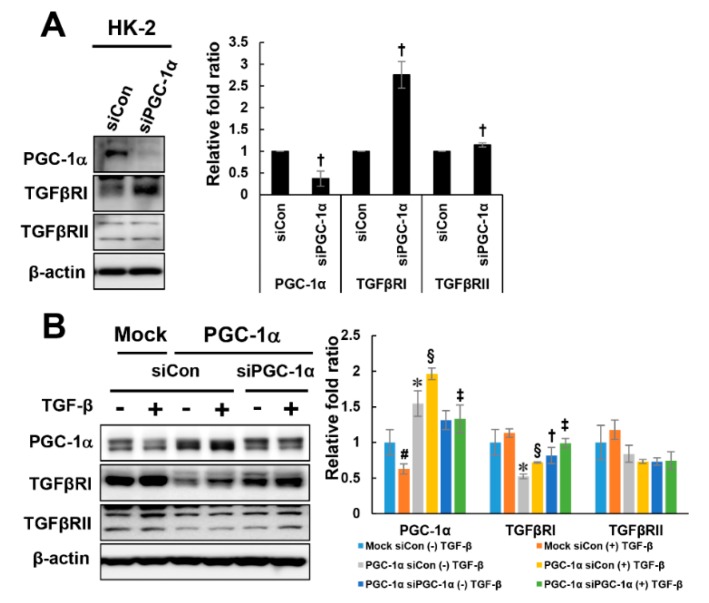
PGC-1α-specific TGFβRI regulation. To confirm whether TGFβRI modulation is PGC-1α-specific, PGC-1α was knocked down using PGC-1α siRNA (30 nM, Dharmacon) in HK-2 cells (**A**) and mock and PGC-1α cells (**B**). After 48 h of transfection, the cells were treated with TGF-β for 2 h, harvested and analyzed by western blotting with the indicated antibodies (against PGC-1α, TGFβRI, and TGFβRII). **^#^** *p* < 0.05, TGF-β-treated vs. untreated mock cells; * *p* < 0.05, untreated mock cells vs. PGC-1α cells; **^§^** *p* < 0.05, TGF-β-treated mock cells vs. PGC-1α cells; ^†^ *p* < 0.05, siCon vs. siPGC-1α treatment of untreated PGC-1α cells; ^‡^ *p* < 0.05, siCon vs. siPGC-1α treatment of TGF-β-treated PGC-1α cells.

To further investigate whether the downregulation of TGFβRI is specific to PGC-1α, PGC-1α siRNAs were used to knock down PGC-1α in HK-2 and PGC-1α cells. Knockdown of endogenous PGC-1α in HK-2 cells increased the expression of TGFβRI, but there was no change in TGFβRII expression by PGC-1α knockdown (Figure 5A). Knockdown of PGC-1α in PGC-1α cells restored the reduced expression level of TGFβRI but did not affect the TGFβRII expression level (Figure 5B). These results suggest that the downregulation of TGFβRI is specific to PGC-1α.

### 2.5. PGC-1α Regulates let-7b/c-Mediated TGFβRI Expression

**Figure 6:**
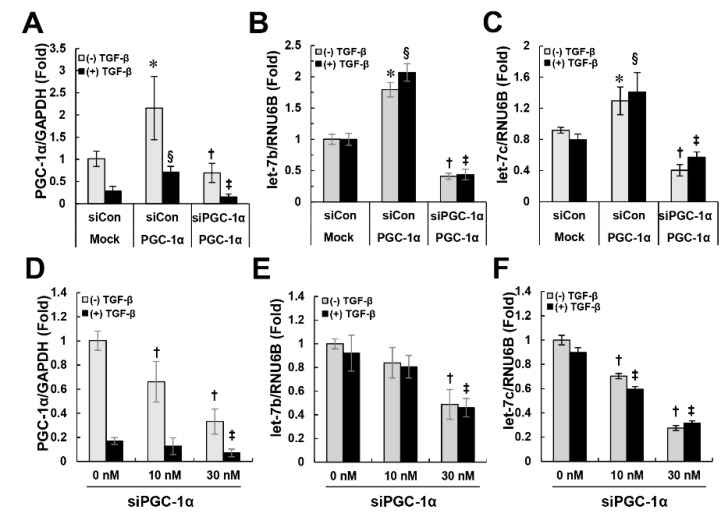
Regulation of *let-7b/c* by PGC-1α. To examine the regulation of *let-7b/c* by PGC-1α, control siRNA- or PGC-1α siRNA-transfected mock and PGC-1α cells (**A**–**C**) and PGC-1α siRNA-transfected HK-2 cells (**D**–**F**) were prepared as described in the Materials and Methods section. The levels of *let-7b, let-7c* (target miRNA) and RUN6B (internal reference) were quantitatively analyzed by real-time PCR. The bar graph shows the relative ratio of *let-7b* or *let-7c* to RUN6B, with the value in untreated mock cells set to 1. * *p* < 0.05, untreated mock cells vs. PGC-1α cells; **^§^** *p* < 0.05, TGF-β-treated mock cells vs. PGC-1α cells; **^†^** *p* < 0.05, siCon vs. siPGC-1α treatment of untreated PGC-1α cells; **^‡^** *p* < 0.05, siCon vs. siPGC-1α treatment of TGF-β-treated PGC-1α cells.

Next, we attempted to elucidate the mechanism by which PGC-1α regulates TGFβRI. We formed a hypothesis based on the following clues. First, this regulatory mechanism of PGC-1α must involve a protective effect against EMT. Second, PGC-1α selectively acts on TGFβRI but not TGFβRII to regulate the activation of Smad2/3, leading to its decreased expression. Third, the regulation of TGFβRI by PGC-1α is regulated at the transcriptional level rather than the translational level. Recent studies have reported that the let-7 family of microRNAs modulate the expression of TGFβRI by binding to the 3’ untranslated region (UTR) of TGFβRI [14,15]. Consistent with this, the level of *let-7b/c* was higher in PGC-1α cells than in mock cells, and when the PGC-1α level was decreased again, the level of *let-7b/c* was lower than that of the mock cells (Figure 6A–C). Knockdown of endogenous PGC-1α in HK-2 cells by a dose-dependent treatment with siPGC-1α also resulted in the dose-dependent downregulation of *let-7b/c* (Figure 6D–F). These results suggest that PGC-1α is involved in the regulation of *let-7b/c*-mediated TGFβRI expression and inhibits subsequent activation of the TGFβRI-mediated Smad2/3 signaling pathway, resulting in protection against EMT.

## 3. Discussion

Mitochondria are power players in kidney function, and their functions are fine-tuned through the modulation of their biogenesis, bioenergetics, dynamics and clearance from cells (mitophagy) [16,17]. Mitochondrial dysfunction plays a crucial role in the pathogenesis of kidney disease and is involved in the physiological process of renal fibrosis [18]. Indeed, transcriptional analysis of biopsy samples from CKD patients showed that the pathways associated with mitochondrial function are downregulated compared to those in healthy individuals [19]. Therefore, attention has been focused on identifying mitochondrial targets as therapeutic agents to improve the mitochondrial dysfunction in CKD patients [20]. We have demonstrated the protective effect of PGC-1α on EMT progression. The decreased expression of PGC-1α was observed in the UUO kidney and TGF-β-treated HK-2 cells, and TGF-β-induced EMT was inhibited in HK-2 cells overexpressing PGC-1α. These findings suggest PGC-1α as an important therapeutic target for CKD.

In this study, we proposed the PGC-1α-induced negative regulation of TGFβRI as a protective mechanism in TGF-β-treated EMT progression. This effect led to the subsequent regulation of the TGF-β/Smad signaling pathway. Interestingly, PGC-1α targeted only TGFβRI as a means to modulate TGF-β/Smad signaling. Research on TGFβRI has also been carried out in different fields [4]. Galunisertib (LY2157299), an oral inhibitor of TGFβRI kinase, inhibited the phosphorylation of Smad2/3 in a liver fibrosis model, thereby suppressing the production and maturation of COL1A1 [21]. The possible antifibrotic effects of galunisertib on liver fibrosis enable its application to kidney fibrotic diseases. As another example of research on TGFβRI in different fields, exercise-trained muscles were shown to release myokine, which is beneficial for CKD patients. In addition, a paper reported crosstalk between the muscle and kidney in muscle-specific PGC-1α-overexpressing mice, which mimicked the effects of strength training. Irisin acts to protect against kidney fibrosis by interfering with TGFβRI activation through binding to TGFβRII [22,23]. As shown by our study, PGC-1α targets and regulates TGFβRI at the transcriptional level as well as TGFβRI downstream signaling.

Finally, we have shown the mechanism by which PGC-1α targets TGFβRI downregulation. PGC-1α regulated the expression of let-7b/c, miRNA for which the 3’ UTR of TGFβRI contains a binding site. In addition, *let-7b/c* was shown to be expressed in a PGC-1α-dependent manner. The level of *let-7b/c* in PGC-1α cells was decreased by siRNA against PGC-1α. *Let-7b/c* is an isotype of the miRNA *lethal-7* (*let-7*) family. In humans, the *let-7* family is composed of nine mature miRNAs (*let-7a, let-7b, let-c, let-7d, let-7e, let-7f, let-7g, let-7i*, and *miR-98*) encoded by 12 different genes [24]. *Let-7* family members play two major biological roles; these miRNAs act as essential regulators of terminal differentiation and as fundamental tumor suppressors [25]. Recently, members of the *let-7* family were suggested to act as an antifibrotic miRNAs in CKD [26,27]. TGF-β downregulated *let-7b* in rat proximal tubular epithelial cells (NRK52E) with the upregulation of TGFβRI, leading to fibrogenesis. Ectopic expression of *let-7b* inhibited Smad3 activity by upregulating TGFβRI expression [15]. *Let-7* family members (*let-7b/c/d/g/i*) mRNAs were also downregulated in glomerular mesangial cells under diabetic conditions. The *Lin28b/let-7* pathway, which is driven by the *Lin28b* upregulation-induced repression of *let-7*, was shown to control TGF-β-induced collagen accumulation in DN [27]. Lipoxins are endogenous lipid mediators that promote the resolution of inflammation and inhibit renal fibrosis [28,29]. Lipoxin A4 (LXA_4_) attenuated renal fibrosis in a rat UUO model and TGF-β-treated HK-2 cells, which was associated with an increased expression of *let-7c* miRNA [14]. As shown by bioinformatic analysis of the cohort of miRNAs regulated by LXA_4,_ TGFβRI and HMGA2 were implicated as targets of *let-7c*. The 3’ UTRs of human TGFβRI and HMGA2 contain two and six predicted *let-7c*-binding sites, respectively. LXA4-mediated upregulation of *let-7c* suppressed the TGF-β signaling pathway members including TGFβRI [14].

**Figure 7:**
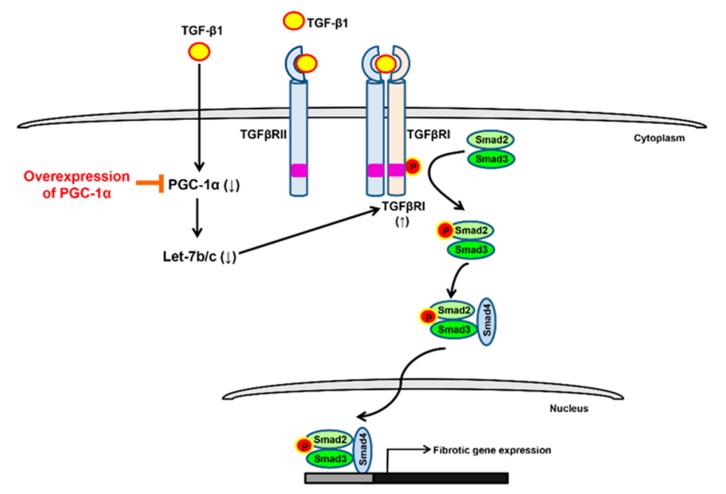
Regulation of *let-7b/c*/TGFβRI/Smad2/3 axis by PGC-1α. Treatment of TGF-β in human proximal tubule epithelial cells lowers the expression of PGC-1α and negatively regulates the expression of its downstream miRNA, *let-7b/c*. Downregulation of *let-7b/c* increases the expression of TGFβRI, which induces phosphorylation of TGFβRI by TGF-β treatment and subsequently increases the activity of Smad2/3. As a result, the EMT-related gene expression is increased and the EMT progression proceeds. The targeting strategy of the *let-7b/c*/TGFβRI/Smad2/3 axis by overexpression of PGC-1a is proposed as one of the therapeutic targets that inhibits EMT progression.

The protective mechanism of PGC-1α against EMT progression can be explained as follows. First, *let-7b/c* acts as a downstream effector of PGC-1α that is positively upregulated by the overexpression of PGC-1α. Second, PGC-1α-mediated *let-7b/c* upregulation effectively inhibits the upregulation of TGF-β-induced TGFβRI. Third, the downregulation of *let-7b/c*-mediated TGFβRI decreases TGF-β-induced Smad2/3 activation. Fourth, the inhibition of TGF-β/Smad signaling lowers the expression of EMT markers and transcription factors, decreasing EMT progression. In conclusion, this study suggests PGC-1α as a candidate for the targeting of TGF-β/Smad signaling. We have shown a novel targeting scheme by which the *let-7b/c*/TGFβRI/Smad2/3 axis is regulated by PGC-1α (Figure 7). However, limitations of this study exist as well. Renal fibrosis is characterized by tubulointerstitial fibrosis, and resident fibroblasts are key players in fibrosis [30]. It may be possible that PGC-1α’s protective effect on EMT progression is part of renal fibrosis. Renal inflammation also plays a central role in the initiation and progression of CKD by causing fibrosis [31]. There are many reports that PGC-1α has an anti-inflammatory effect [32,33]. Therefore, it is necessary to investigate whether the inhibition of progression to EMT by TGF-β treatment in PGC-1α cells is an indirect effect of inflammation modulation. Finally, further research is needed to determine whether the effects of PGC-1α will be equivalent to the effects of *NRF-1/2*, *ERRα,* and *PPARγ*, known as target transcription factors of PGC-1α [34]. In vivo experiments using known pharmacological inducers of PGC-1α are necessary to determine the inhibitory effects of PGC-1α on EMT progression and the regulatory mechanisms of *let-7b/c*/TGFβRI/Smad2/3 axis [35,36,37,38].

## 4. Materials and Methods

### 4.1. Reagents and Antibodies

Recombinant human TGF-β1 was purchased from R&D Systems (Minneapolis, MN, USA). Fugene HD transfection reagent was from Promega (Madison, WI, USA). Antibodies against PGC-1α and fibronectin were from Santa Cruz (Dallas, TX, USA) or Abcam (Cambridge, MA, USA). Antibodies against phospho-Stat3 (Tyr705), Total-Stat3, Total-Smad2/Smad3, Phospho-Smad2 (Ser465/467)/Smad3 (Ser423/425), TGF-β, Total-Jak2, and Phospho-Jak2 were all from Cell Signaling Technology (Danvers, MA, USA). E-cadherin was purchased from BD Biosciences (Franklin Lakes, NJ, USA). Antibodies against α-SMA and β-actin were from Sigma-Aldrich (St. Louis, MO, USA). The selective antibiotics, zeocin was purchased from Invitrogen (Carlsbad, CA, USA).

### 4.2. UUO Model

The animal experiments were approved by the Animal Care Regulations (ACR) Committee of Chonnam National University Medical School (CNU IACUC-2016-50, date of approval 20 December 2016) and our protocols conformed to the institution guidelines for experimental animal care and use. Experiments were performed using male C57BL/6 mice (18 ~ 20 g, Samtako Bio Inc., Osan, Korea). Mice were housed under controlled temperature (21 ± 2 °C) in a 12 h light-dark cycle. Unilateral ureteral obstruction (UUO) was induced by ligation of the left ureter for one week (*n* = 8) or for two weeks (*n* = 8). The abdominal cavity was opened, and 2–0 silk ligature was placed at left proximal ureter under anesthesia with ketamine (50 mg/kg, intraperitoneally; Yuhan, Seoul, Korea). The control group for one week (*n* = 8) and two weeks (*n* = 8) received the same treatment, with the exception of the ligature.

### 4.3. Cell Culture and TGF-β Treatment

HK-2 cells (ATCC, Manassas, VA, USA), were cultured in complete DMEM-F12 media (WelGene, Daegu, Korea) supplemented with 10% fetal bovine serum, 50 U/mL penicillin and 50 μg/mL streptomycin at 37 °C under a humidified 5% CO_2_ atmosphere. Stably transfected HK-2 cells ectopically expressing indicated constructs (Mock and PGC-1α) were maintained in complete medium containing 200 µg/mL of zeocin (Invitrogen, Carlsbad, CA, USA) [13]. For TGF-β treatment, cells were starved for one day with serum free media and treated with 10 ng/mL of TGF-β for the indicated time.

### 4.4. Transfection of siRNA

For knock-down of PGC-1α, PGC-1α siRNA (ON-TARGETplus SMARTpool, Dhamacon, Cat# L-005111-00-0005) and control siRNA (ON-TARGETplus Non-targeting Control pool, Dhamacon, Cat# D-001810-10-05) were transfected into HK-2 cells at 60% confluence using DhamaFect 1 solution at a final concentration of 10 or 30 nM for 24 h. Cells were starved for one day with serum free media and then stimulated with TGF-*β*1 as previously described.

### 4.5. Quantification of mRNA and miRNA

To quantify mRNA levels, total RNA was extracted from mouse kidney and HK-2 cells using TRIzol reagent (Invitrogen). cDNA was then reverse transcribed from 1 μg sample of total RNA using QuantiTect Reverse Transcription Kit (Qiagen Science, Germantown, MD, USA). Real-time PCR was performed using SYBR Green PCR master mix (Thermo Fisher Scientific, Austin, Texas, USA) and StepOnePlus Real-Time PCR System (Thermo Fisher Scientific, Austin, Texas, USA). Primer sequences for real-time PCR were as follows: hPGC-1α, forward, 5′-TCTCAGTACCCAGAACCATGCA-3′, reverse, 5′-GCTCCATGAATTCTCAGTCTTAACAA-3′; hGAPDH, forward, 5′- GACATCAAGAAGGTGGTGAA-3′, reverse, 5′- TGTCATACCAGGAAATGAGC-3′. hTGFβRI (Cat# HP100487) and hTGFβRII (Cat# HP100394) were purchased with Sino Biological Inc (Beijing, China). miRNA-enriched RNA was extracted from HK-2 cells using a miRNeasy RNA extraction kit Kit (Qiagen Science, Germantown, MD, USA). Total RNA containing miRNA (0.5 to 1 μg) was converted into cDNA using miScript II RT Kit Kit (Qiagen Science, Germantown, MD, USA). Real-time PCR was performed using miScript SYBR Green PCR Kit with *let-7b* (Hs_let-7b_1 miScript Primer Assay, Cat# MS00003122)- or *let-7c* (Hs_let-7c_1 miScript Primer Assay, Cat# MS00003129)-specific miScript Primer Assay and Universal Primer. The human RNU6B miScript Primer Assay was used as control miRNA.

### 4.6. Immunofluorescence (IF) Staining

The mock and PGC-1α cells were seed onto four well-cell culture slides (2 X 10^4^/well) and were proceed as mentioned previous. Cells were washed with phosphate-buffered saline (PBS) and were fixed in 4% paraformaldehyde for 10 min. Subsequently, cells were permeabilized with permeabilization buffer (0.5% Triton X-100 in PBS) and the slides were incubated with primary antibodies to α-SMA (1/1000 dilution), E-cadherin (1/50 dilution), and PGC-1α (1/300 dilution, Abcam Cat# ab54481) in diluted with equilibration buffer (1% bovine serum albumin, 0.5% Triton X-100 in PBS) at 4 °C overnight. Following incubation with primary antibody, the cells were washed with equilibration buffer and incubated for 1 h at room temperature with anti-mouse-FITC (for α-SMA and E-cadherin), and anti-rabbit-Cy3 (For PGC-1α) conjugated secondary antibodies (Vector Lab, Burlingame, CA, USA). The nuclei were counterstained using SlowFade Gold antifade reagent with DAPI (Invitrogen, Carlsbad, California, USA). Images were captured using a confocal microscope (LSM 510; Carl Zeiss, Oberkochen, Germany). Image was magnified at 800×, Bar = 20 μm.

### 4.7. Statistical Analysis

Values are presented as mean ± standard deviation (S.D.). Between-group differences were measured using one-way ANOVA with post-hoc Tukey HSD (Honestly Significant Difference) analysis where appropriate. *p*-values < 0.05 were considered as statistically significant. All experiments were performed at least three times or more.

## Supplementary Materials

Supplementary materials can be found at https://www.mdpi.com/1422-0067/20/20/5084/s1.
