# Supplementary material for: PGC-1α Suppresses the Activation of TGF-β/Smad Signaling via Targeting TGFβRI Downregulation by let-7b/c Upregulation
Source: Int J Mol Sci. 2019 Oct 14;20(20):5084. doi: 10.3390/ijms20205084 (PMC6829475; doi:10.3390/ijms20205084)
Supplement: Supplementary file 1 [file ijms-20-05084-s001.zip › ijms-596913-SI.pptx]

## Slide 1
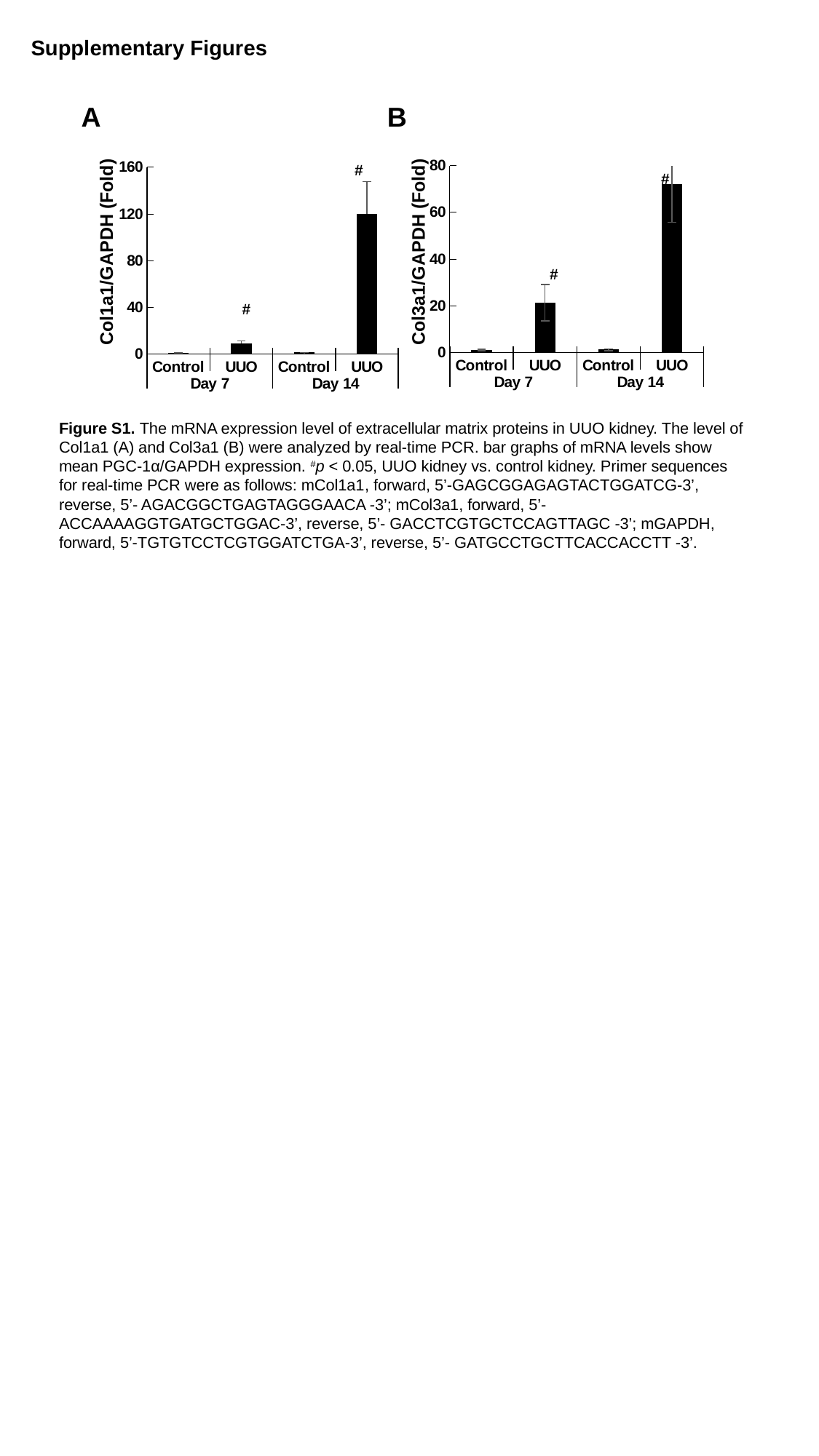

Supplementary Figures
A
B
### Chart
| Category | Col3a1 |
|---|---|
| Control | 1.0427805517170852 |
| UUO | 21.296688900591125 |
| Control | 1.187247501578106 |
| UUO | 72.04764706248058 |
### Chart
| Category | Col1a1 |
|---|---|
| Control | 1.0104131009144426 |
| UUO | 8.864873049527546 |
| Control | 1.0978662287175824 |
| UUO | 119.84474494972342 |#
#
Col1a1/GAPDH (Fold)
Col3a1/GAPDH (Fold)
#
#
Figure S1. The mRNA expression level of extracellular matrix proteins in UUO kidney. The level of Col1a1 (A) and Col3a1 (B) were analyzed by real-time PCR. bar graphs of mRNA levels show mean PGC-1α/GAPDH expression. #p < 0.05, UUO kidney vs. control kidney. Primer sequences for real-time PCR were as follows: mCol1a1, forward, 5’-GAGCGGAGAGTACTGGATCG-3’, reverse, 5’- AGACGGCTGAGTAGGGAACA -3’; mCol3a1, forward, 5’-ACCAAAAGGTGATGCTGGAC-3’, reverse, 5’- GACCTCGTGCTCCAGTTAGC -3’; mGAPDH, forward, 5’-TGTGTCCTCGTGGATCTGA-3’, reverse, 5’- GATGCCTGCTTCACCACCTT -3’.
